# Comparison of volatile organic compounds in different parts of *Houttuynia cordata* Thunb. via E-nose, GC–IMS, and multivariate statistical analysis

**DOI:** 10.3389/fnut.2026.1861403

**Published:** 2026-07-24

**Authors:** Jincheng Zhao, Manshu Zou, Qianqian Luo, Jingyi Tang, Chang Lei

**Affiliations:** 1 State Key Laboratory of Chinese Medicine Powder and Medicine Innovation in Hunan (Incubation), Academy of Chinese Medical Sciences (Science and Technology Innovation Center), Hunan University of Chinese Medicine, Changsha, China; 2 Traditional Chinese Medicine Prevention and Treatment of Depressive Diseases, Changsha, China

**Keywords:** different parts, gas chromatography–ion mobility spectrometry (GC-IMS), Heracles NEO ultra-fast gas-phase electronic nose, *Houttuynia cordata* Thunb., multivariate statistical analysis, volatile organic compounds

## Abstract

**Introduction:**

*Houttuynia cordata* Thunb. (*H. cordata*) is a well-known medicinal and edible plant extensively utilized in Asia. The different parts of *H. cordata,* including its leaves, stems, and rhizomes, possess distinct functionalities and clinical applications.

**Methods:**

This study systematically compared and analyzed the composition of volatile organic compounds (VOCs) in three different parts of *H. cordata* using gas chromatography-ion mobility spectrometry (GC-IMS) and Heracles NEO ultra-fast gas phase electronic nose (E-nose) complemented by multivariate statistical analysis.

**Results:**

The results from E-nose and GC-IMS revealed significant variations in the composition and peak intensity of VOCs among the different parts of *H. cordata*. Notably, the differences in VOCs and odor between the leaves and stems were minimal. 63 signal peaks were detected, and 55 VOCs were tentatively identified via GC-IMS, with terpenes constituting the largest proportion. Through the application of partial least squares discriminant analysis (PLS-DA), cluster analysis (CA), and the variable importance in projection (VIP) method for feature selection, 16 VOCs were identified as capable of effectively distinguishing the different parts of *H. cordata.*.

**Conclusion:**

This study demonstrated the effectiveness of combining GC-IMS with E-nose technology to detect VOCs in different parts of *H. cordata*, establishing a theoretical basis for resource development.

## 1 Introduction

*Houttuynia cordata* Thunb. (*H. cordata*) is a medicine food homology plant, used widely as Chinese herbal medicine and food. It is broadly distributed across Asia, including China, South Korea, Japan, Thailand, and India (1). In China, it is commonly called *“yuxingcao,”* due to fish-like odor characteristic of leaves, stems, and rhizomes. The plant is rich in bioactive compounds, including volatile organic compounds (VOCs), flavonoids, phenolic acids, terpenes, and alkaloids (2). Notably, VOCs exhibit various pharmacological activities, such as antibacterial, anti-inflammatory, anticancer, antifungal, and antiviral properties (3, 4). In 2002, *H. cordata* was officially recognized by the Chinese National Health Commission as a food and medicine substance, and approved for COVID-19 treatment in 2020 (5).

*H. cordata* exhibits notable differences in flavor characteristics, health benefits, and nutritional components between aboveground parts (leaves and stems) and underground rhizomes (1). According to the *Chinese Pharmacopoeia,* both the fresh whole plant and the dried aboveground parts are sanctioned for medicinal application. In contrast, all parts of *H. cordata* are considered edible when utilized as food. The underground rhizomes are particularly valued in southwestern China, where they are commonly consumed as a vegetable or condiment. These rhizomes are characterized by a mild fishy odor, a crisp and tender texture, and a juicy quality with a slightly sweet aftertaste. They are recognized for their therapeutic effects, including heat-clearing, detoxification, abscess elimination, and pus drainage, and are well known as “Traditional Chinese Medicine Antibiotic.” The aboveground stems of *H. cordata* are favored by consumers as sprout vegetables due to their crispness, distinctive flavor, and palatability. Furthermore, when fresh *H. cordata* leaves are crushed, they release a significant amount of volatile oil. This oil has a strong pungent and metallic, and the leaves are tender and juicy, making them suitable for extracting food-grade spices. At present, processed products derived from *H. cordata*, such as ultrafine powder, extracts, and tea bags, are often plagued by issues of mixing or adulteration involving different parts in the market. These issues adversely affect quality and safety, and harming consumer interests. Therefore, it is of practical significance to distinguish and identify different parts of *H. cordata* using an efficient method. This will enhance the efficient utilization of the entire plant and ensure safety and effectiveness of its derived products.

Odor, resulting from the combination of VOCs, is a key quality indicator of traditional Chinese medicine. Traditional sensory evaluation is difficult to objectively quantify odor characteristics due to inter-individual differences in olfactory sensitivity and susceptibility to olfactory fatigue. Gas chromatography-ion mobility spectrometry (GC-IMS) is a fast analytical method for detecting VOCs, combining the high-efficiency separation capability of gas chromatography (GC) with the high sensitivity of ion mobility spectrometry (IMS). This method is particularly well-suited for the rapid identification and dynamic monitoring of VOCs (6). It provides intuitive spectral visualization, straightforward operation, rapid response, and requires no complex sample preparation. The electronic nose (E-nose) is an analytical device designed for odor analysis, enabling the recognition, analysis, and detection of volatile substances by simulating the olfactory mechanisms of biological organisms. The Heracles II Neo Ultra-Fast Gas Phase Electronic Nose is a sophisticated device for odor detection, which integrates the functionalities of both GC and E-Nose technologies. It uses a dual-column chromatographic system for quick and thorough odor analysis, offering easy operation, high objectivity, sensitivity, fast response, and excellent repeatability. The integration of E-Nose and GC-IMS can enhance the instrument’s ability to deliver comprehensive and reliable odor information. It is widely used in various fields like food (7), tea (8), medicine (9) and agriculture (10). Additionally, it plays a crucial role in the authentication of Chinese medicinal materials and medicinal food sources (11), classification of plant parts (12), authenticity evaluation (13), and the optimization of methods (14). Furthermore, multivariate statistical techniques such as Principal Component Analysis (PCA), Euclidean Distance (ED), Partial Least Squares-Discriminant Analysis (PLS-DA), and Cluster Analysis (CA) (15–17), are highly effective for processing complex multivariable datasets. They simplify data by reducing redundant indicators to key components and enable efficient analysis using advanced software. This approach allows for more comprehensive and accurate quality control of traditional Chinese medicine (18).

So far, the research of *H. cordata*’s different parts has predominantly concentrated on chemical composition and pharmacological activity studies (1, 19–21). Although some investigations have reported on the volatile component composition of different parts of *H. cordata*, these investigations primarily emphasize comparisons of essential oils or different extraction methods. They have not utilized the method of GC-IMS and E-Nose technology. This study utilized GC-IMS and E-nose to analyze and preliminarily infer the VOCs and odors from three different parts (leaves, stems, and rhizomes) of *H. cordata*, and examined the differences among these parts. Through the application of multivariate statistical analyses, including PCA, ED, PLS-DA, and CA, we established the fingerprints and identified the potential characteristic VOCs. The results of this study are helpful for providing a theoretical foundation for the identification and utilization of resources from different parts of *H. cordata.*

## 2 Materials and methods

### 2.1 Materials

**Figure 1:**
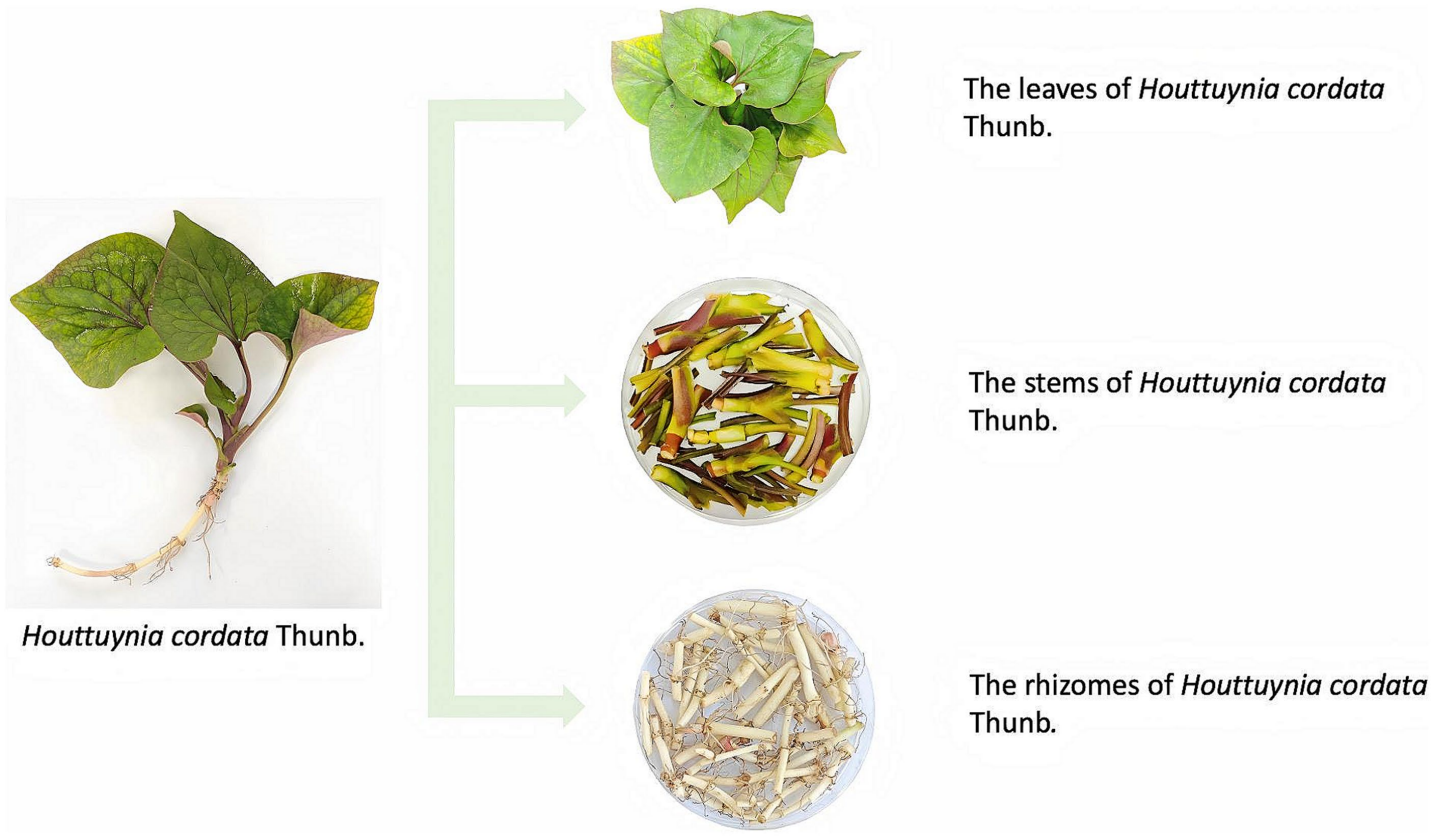
The whole plant and different parts of *H. cordata*.

Fresh samples of *Houttuynia cordata* Thunb. were collected from Tashui, Anzhou, Mianyang, Sichuan, China (31.46437899°N, 104.4207691°E), kept in ice-filled containers, and transported to the laboratory within 48 h. The entire plants were thoroughly cleaned to surface impurities and soil, and subsequently dissected into three parts: leaves (named YXCY), stems (named YXCJ), and rhizomes (named YXCGZJ), as shown in Figure 1.

### 2.2 Heracles neo ultra-fast gas phase electronic nose analysis

The samples were analyzed using a Heracles Neo Ultra-Fast Gas Phase Electronic Nose (Alpha MOS, Toulouse, France), equipped with an MXT-5 low-polarity column and an MXT-1701 medium-polarity column. 3 g samples (YXCY, YXCJ, and YXCGZJ) was accurately weighed, respectively, placed in a 20 mL headspace bottle and sealed with a PTFE gasket. Five independent biological replicates of each sample were analyzed. The incubation conditions were maintained at 60 °C for 15 min, with the injection port temperature set at 200 °C. A 5000 μL sample was injected at a rate of 250 μL/s for 25 s, with a split ratio of 10 mg/mL. The trap temperature was initially set at 40 °C, then increased to 240 °C and held for 30 s. The column temperature was initially held at 40 °C for 30 s, followed by a temperature gradient of 1.5 °C/s to 120 °C, 0.3 °C/s to 140 °C, and 2.0 °C/s to 250 °C, resulting in a total acquisition time of 290 s. The detector temperature was set at 260 °C, with a FID gain of 12.

### 2.3 GC-IMS analysis

The VOCs of *H. cordata* from different parts were analyzed using GC-IMS (Flavourspec^®^, G. A. S, Germany) and a CTC-PAL 3 automatic sampling device (CTC Analytics AG, Switzerland). 0.5 g of samples (YXCY, YXCJ, and YXCGZJ) was accurately weighed, respectively, and placed in a 20 mL headspace bottle, incubated at 40 °C for 15 min. Three independent biological replicates of each sample were analyzed. A 500 μL aliquot of the sample was injected at a temperature of 85 °C and a speed of 500 rpm for a duration of 15 min. Nitrogen (purity ≥ 99.999%) was used as both the carrier and drift gas. The gas chromatography column was maintained at 60 °C, with a drift flow rate of 75 mL/min. The initial flow rate was set at 2.0 mL/min for 2 min, then increased linearly to 10.0 mL/min over 8 min, and subsequently to 100 mL/min over 10 min, before being maintained for 30 min. The injection temperature was set at 80 °C, and the total run time was 50 min. The ion mobility spectrometry (IMS) was conducted using tritium (^3^H) in positive ion mode. The transfer tube had a length of 53 mm and was maintained at a temperature of 45 °C. The analysis used an MXT-WAX capillary column (15 m × 0.53 mm, 1.0 μm, Restek, Mount EI, USA).

### 2.4 Statistical analysis

VOCs were tentatively identified using the NIST 2020 database, IMS drift time database, and G.A.S. data processing software (version 0.4.10, G.A.S.). Three-dimensional (3D) spectrum, two-dimensional (2D) spectrum, comparative spectrum, fingerprint, PCA were created by the VOCal plug-in data-processing software, such as Reporter (version 11.x), Gallery Plot (version 1.1.0.2), and Dynamic PCA (version 0.0.3). SIMCA software (version 14.1) was used for PLS-DA to calculate the PCA score plot and variable important projection (VIP) value. CA was performed with TB tools-II (version 2.400). Discriminant function analysis (DFA) and compound identification were automatically generated by the integrated systems of each respective analytical technology. Furthermore, this study had not yet established standardized calibration curves for each volatile component, making GC-IMS and electronic nose detection signals insufficient for determining absolute content and precise concentrations. The signal strength data were only useful for comparing relative differences within groups, not for quantifying absolute content. Compound identification using GC-IMS should be regarded as putative rather than definitive when based on database comparisons, considering the methodological limitations and the absence of standardized reference substances. But these limitations did not affect the validity and reliability of the research results.

## 3 Results

### 3.1 E-nose analysis

#### 3.1.1 Gas chromatogram analysis

**Figure 2:**
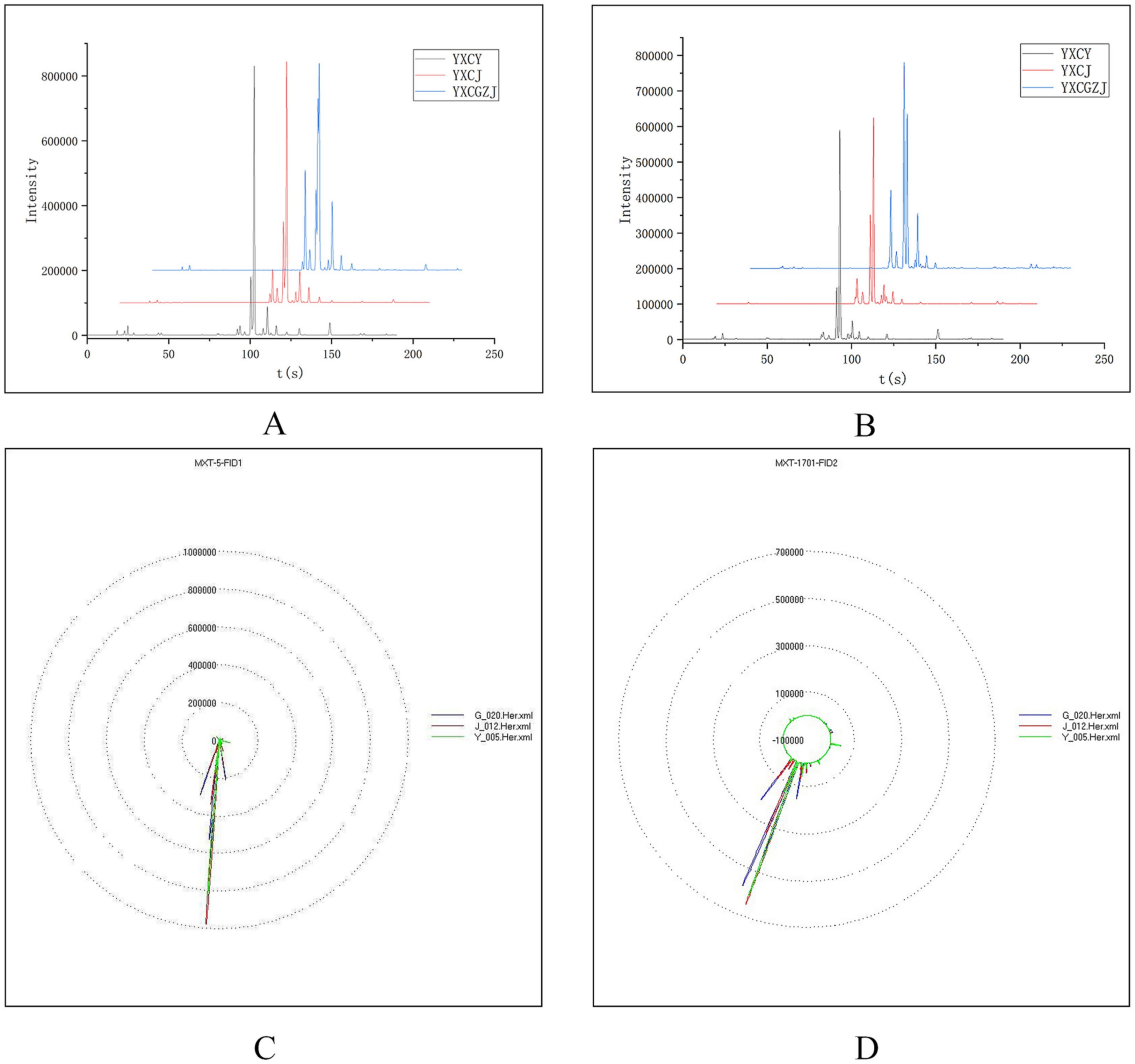
Overlay chromatograms **(A,B)** and radar charts **(C,D)** of MXT-5 or MXT-1701 from different parts of *H. cordata*.

The analysis showed significant differences in retention times and peak areas among samples from different parts of *H. cordata*. Overlaid original gas chromatograms in Figures 2A,B revealed substantial similarity in detection results between the MXT-5 and MXT-1701 columns, especially major peak intensities. However, retention times and peak profiles varied markedly across the three parts. Such variations suggest distinct odor fingerprints and VOC profiles for each part.

Detection results radar charts in Figures 2C,D showed peak areas data in the inner circle. On the MXT-5 column, *H. cordata* stems generated the strongest VOCs response. In contrast, leaves and rhizomes exhibited weaker and more clustered sensor responses, suggesting greater similarity in their VOCs compositions and odor profiles. The MXT-1701 column yielded strong sensor responses for both stems and rhizomes. Leaves produced weaker responses with divergent sensor directions, indicating greater diversity in VOC composition and more complex odor profiles. Overall, distinct odor fingerprint differences were observed for different *H. cordata* parts, reflecting variations in VOCs composition, signal response, or category.

#### 3.1.2 PCA and DFA

**Figure 3:**
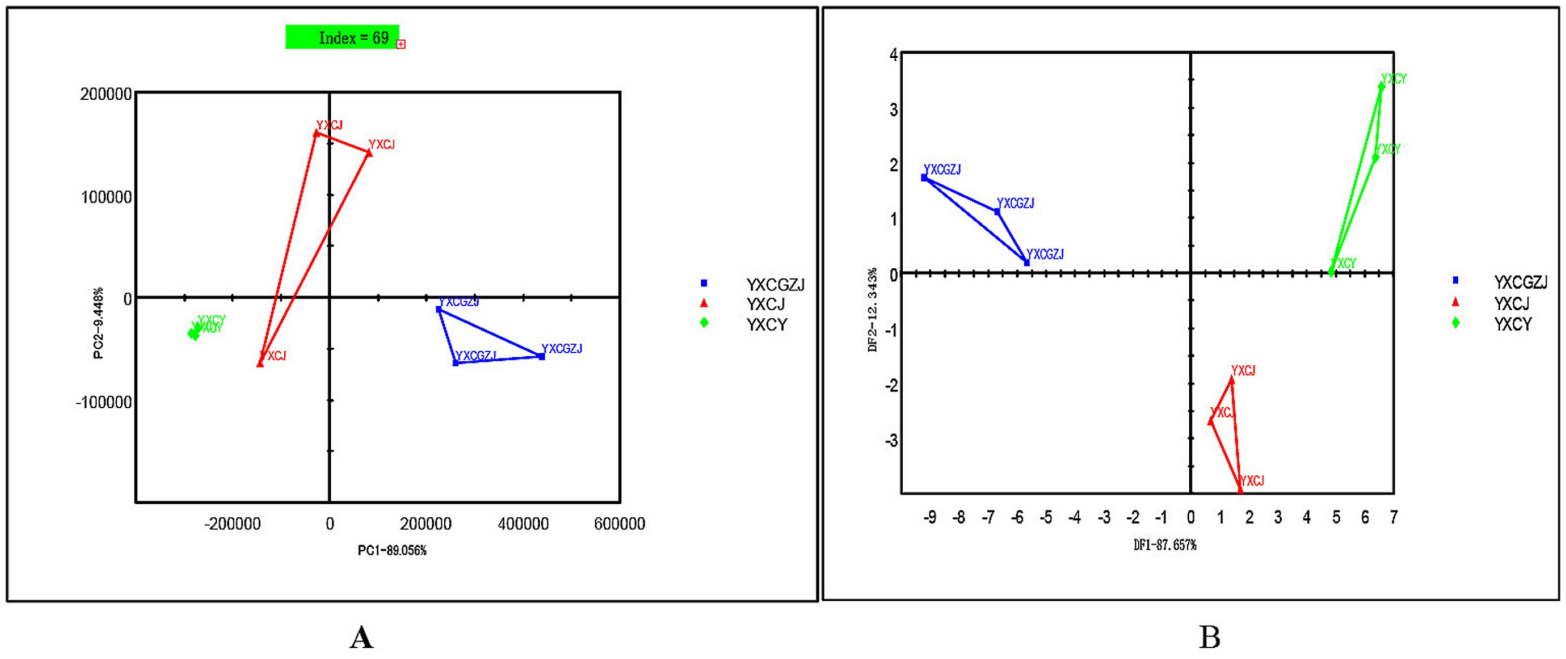
PCA **(A)** and DFA **(B)** from different parts of *H. cordata*.

PCA was performed on the VOCs data from three parts of *H. cordata* samples (Figure 3A). The first two principal components (PC1 and PC2) accounted for 98.504%, effectively capturing most of the odor-related information and revealing clear spatial separation among sample points. This indicates substantial overall odor differentiation among the different parts of *H. cordata*. The position distributions of YXCJ and YXCY are the most similar, suggesting relatively small overall odor difference between these two sample groups. DFA can enhance sample discrimination by minimizing intra-class distances and maximizing inter-class distances. It can address PCA’s limitation in directly predicting categories. This analysis examined odor differences among the three parts of *H. cordata* (Figure 3B). The first two discriminant functions (DF1 and DF2) cumulatively contributed 100%, indicating excellent discriminative performance and effective sample differentiation. In this figure, leaves are positioned near stems, aligning with PCA results.

#### 3.1.3 Compound identification

This study tentatively identified 28 VOCs in different parts of *H. cordata* by comparing chromatographic peaks with the AroChemBase database. As shown in Table 1, the table includes odor thresholds, indicating odor intensity (air medium: mg/m^3^, water medium: mg/kg), with lower thresholds indicating stronger odors. The odor activity value, calculated from relative abundance and odor threshold, helps adjust and trace the sample’s odor. Table 1 also lists average peak areas of PRA samples at various retention times using MXT-5 or MXT-1701 columns. Higher peak areas, detected by FID and mass detectors, indicate higher substance signal response.

**Table 1:** Differential chromatographic peak qualitative results and peak intensity table.

Compound	MXT-5		MXT-1701		Odor Description	Odor threshold	Average peak area		
	tR/s	RI	tR/s	RI			Leaf	Stem	Rhizome
Methyl formate	18.3	379	18.8	467	Agreeable, Fruity, Plum	3e+3(air)	4,535	2,599	3,572
Methanethiol	22.9	449	19.3	476	Cabbage, Cabbage (cooked), Cheese, Egg (rotten), Fishy, Garlic, Gasoline, Meaty, Pungent, Rotten, Sulfurous, Unpleasant	5e-4(air)	8,432	5,169	8,632
Propanal	24.9	479	23.6	560	Acetaldehyde, Cocoa, Earthy, Etheral, Nutty, Plastic, Pungent, Solvent	3e-2(air)	11,339	963	0
acetonitrile	28.5	534	31.6	638	Aromatic, Etheral, Sweet	2e+2(air)	2,832	0	0
2-Methyl-1-propanol	36.2	618	45.7	723	Alcoholic, Bitter, Chemical, Fusel, Glue, Leek, Licorice, Musty, Oil, Solvent, Sweet, Unpleasant, Winey	2e+0(air)	1,146	0	0
(E)-but-2-enal	43.6	658	49.8	746	Floral, Green, Plastic, Pungent	4e-1(air)	3,360	0	0
Trometamol	45.4	668	50.8	751	Characteristic	-	3,094	0	0
3-Ethylheptane	81.2	866	71.7	876	-	-	1909	1,190	1748
1R-(+)-alpha-pinene	92.2	933	82.2	948	Aromatic, Harsh, Minty, Pine, Terpenic	2e-2(air)	13,070	20,532	20,704
1S-(−)-a-pinene	93.7	942	83.3	955	Fresh, Herbaceous, Pine, Resinous, Sharp, Terpenic, Turpentine, Warm	2e+0(air)	18,506	93,216	218,272
3-Ethyloctane	96.6	959	86.6	979	-	-	6,196	42,679	58,071
Tetraethoxysilane	100.5	983			Ester, Faint, Sharp	4e+2(air)	123,342	201,003	209,958
decadiene-1,9	101.6	990	91.2	1,011	-	-	104,452	215,272	470,851
Myrcene	102.5	995	93.1	1,024	Balsamic, Etheral, Fruity, Geranium, Lemon, Metallic, Musty, Plastic, Pleasant, Resinous, Soapy, Spicy, Sweet, Woody	4e-2(air)	554,595	689,391	793,957
1-Methyl-4-isopropenyl-1-cyclohexene	108	1,024	97.8	1,054	Citrus, Etheral, Fruity, Green, Lemon, Licorice, Orange, Pleasant	-	15,242	25,885	25,866
4-Methylhexan-1-ol	-	-	99.2	1,064	Grassy, Sweaty	-	8,150	42,908	112,377
L-Limonene	110.6	1,038	100.5	1,072	Citrus, Minty, Orange, Pine, Terpenic, Woody	1e+1(air)	68,619	95,413	166,755
Butylbenzene	112.8	1,049	103	1,088	-	2e+0(air)	4,769	2,780	2,109
gamma-Terpinene	116	1,066	104.5	1,098	Citrus, Etheral, Fruity, Gasoline, Herbaceous, Lemon, Oily, Sweet, Terpenic, Turpentine, Woody	6e+1(air)	22,819	38,913	39,182
2-Isopropyl-3-methoxypyrazine	122.4	1,099	109.8	1,126	Beany, Bell pepper, Dry, Earthy, Grassy, Green, Pea	3e-6(air)	8,264	15,635	18,417
1-Octanethiol	130.1	1,131	121	1,183	Mild, Sulfurous	1e-4(air)	18,178	8,164	1,365
diterpene	139.4	1,171	145.1	1,285	Cool, Mentholic, Peppermint, Woody	5e-1(air)	0	0	3,322
Decanal	148.9	1,212	151.2	1,313	Aldehydic, Burnt, Citrus, Fatty, Floral, Green, Herbaceous, Lemon, Orange, Orange peel, Soapy, Stew, Sweet, Tallowy, Waxy	3e-3(air)	49,003	3,888	1736
Undecan-2-one	167.8	1,299	166.6	1,388	Creamy, Dusty, Fatty, Floral, Fresh, Fruity, Green, Ketonic, Musty, Orange, Orris, Rose, Strong, Tallowy, Vegetable, Waxy	2e+0(air)	3,286	9,940	16,388
Anethole	169.9	1,313	171	1,416	Anise, Herbaceous, Licorice, Oil, Spicy, Sweet	5e-2(water)	3,513	851	0
Butylate	183.6	1,415	183.2	1,520	Aromatic	-	2,402	0	0
alpha-Himachalene	187.3	1,449	179.8	1,488	-	-	0	1,332	1970
Methyl dodecanoate	195.3	1,531	191.9	1,615	Coconut, Creamy, Fatty, Floral, Fruity, Mushroom, Soapy, Sweet, Waxy, Waxy (weak)	2e-3(air)	2,839	2,896	2,644

**Figure 4:**
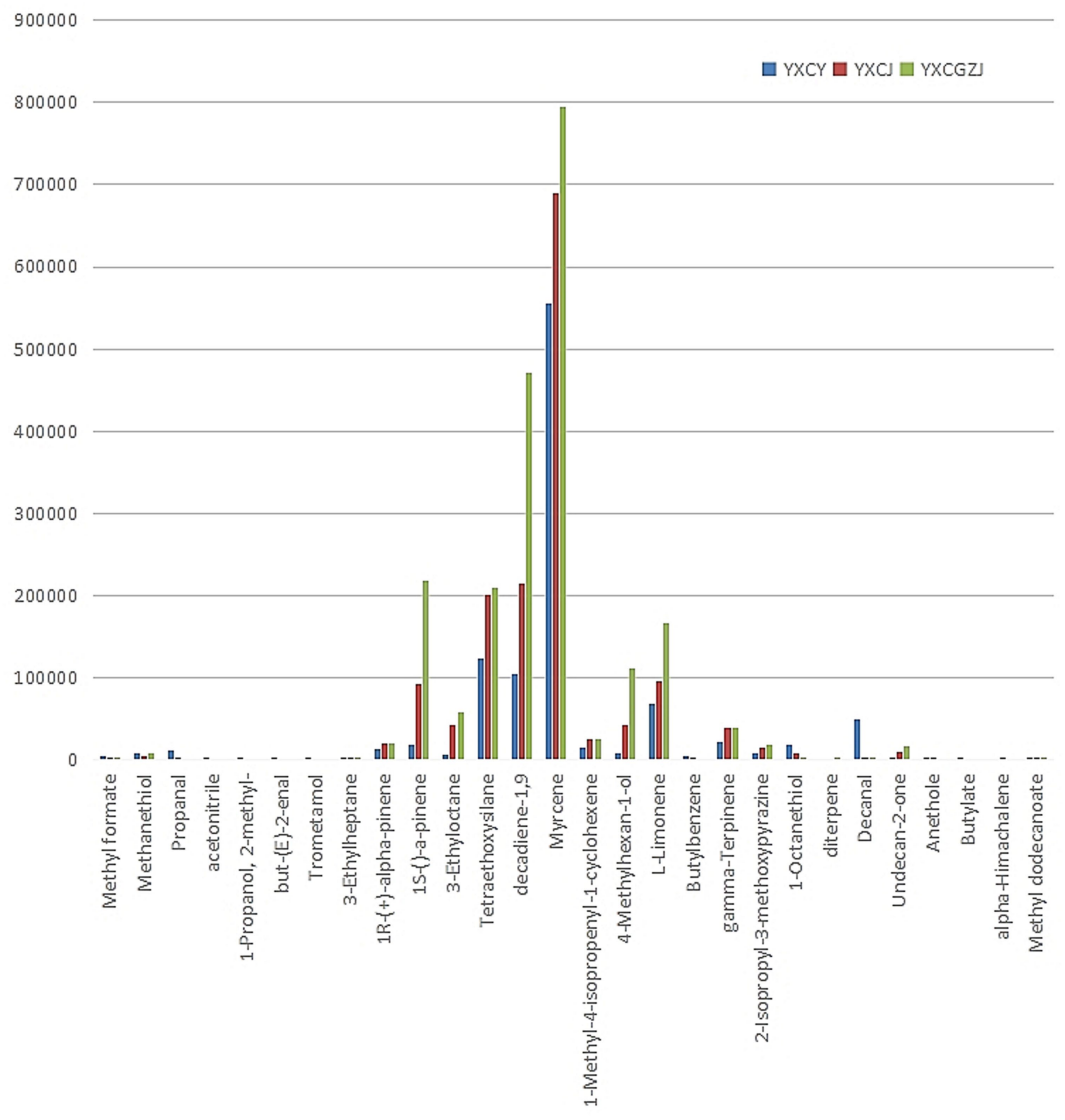
Bar chart of differential compound peak intensity from different parts of *H. cordata*.

To visually compare VOCs variations in different parts of *H. cordata*, a histogram was constructed (Figure 4). The histogram displays VOCs on the x-axis and average peak area on the y-axis. The number of VOCs types tentatively detected in various parts of *H. cordata* followed a decreasing order: leaves (26 types) > stems (22 types) > rhizomes (21 types). Myrcene was consistently detected across all three parts and exhibited the highest peak intensity. The leaves of *H. cordata* were characterized by elevated levels of 1-Octanethiol and Decanal relative to the stems and rhizomes, contributing to a slightly spicy aroma with earthy and cocoa undertones. In the rhizomes, terpenoids and esters were predominant, imparting a slightly cool odor with minty and woody notes. Stems exhibited a moderate diversity and abundance of VOCs. This compositional balance produced a relatively neutral odor, blending the pungency characteristic of the leaves with the woody notes of the rhizomes.

### 3.2 GC-IMS analysis

#### 3.2.1 Qualitative analysis of VOCs in different parts of the *Houttuynia cordata* samples

GC-IMS was used in combination with the NIST and IMS databases, integrating retention index, retention time, and drift time data to analyze and verify signal peaks for VOCs detection in different parts of *H. cordata*. A total of 63 signal peaks were detected, and 55 VOCs were tentatively identified, including 16 terpenoids, 9 ketones, 9 alcohols, 8 aldehydes, 5 esters, and 3 organic acids. Additionally, 8 VOCs remained unidentified and were numbered 1 to 8, as shown in Table 2.

**Table 2:** Detailed list of VOCs in three different parts of *H. cordata*.

Compound	CAS#	Formula	MW	Odor Description	RI	Rt/s	Dt/ms
(R/S)-linalool	78–70-6	C_10_H_18_O	154.3	citrus, rose, woody, blueberry	1570.4	1106.032	1.22407
Propanoic acid	79–09-4	C_3_H_6_O_2_	74.1	yogurt, vinegar	1564.9	1093.676	1.10741
Acetic acid-M	64–19-7	C_2_H_4_O_2_	60.1	spicy	1478.2	915.950	1.05404
Acetic acid-D	64–19-7	C_2_H_4_O_2_	60.1	spicy	1478.2	915.950	1.15387
(Z)-3-hexen-1-ol	928–96-1	C_6_H_12_O	100.2	green, herb	1400.8	781.808	1.23405
1-nonanal	124–19-6	C_9_H_18_O	142.2	rose, citrus, strong oily	1403.8	786.584	1.47325
1-Hydroxy-2-propanone	116–09-6	C_3_H_6_O_2_	74.1	pungent, caramel, fresh	1319.5	662.022	1.06137
1-octanal	124–13-0	C_8_H_16_O	128.2	aldehyde, waxy, citrus, orange, fruity, fatty	1305.7	643.587	1.40804
alpha-terpinolene	586–62-9	C_10_H_16_	136.2	Fresh, woody, sweet, pine, citrus	1293.2	626.954	1.21484
Acetic acid hexyl ester	142–92-7	C_8_H_16_O_2_	144.2	fruity, green, apple, banana, sweet	1288.4	618.055	1.38526
beta-ocimene	13,877–91-3	C_10_H_16_	136.2	floral, woody, fruity	1264.2	574.791	1.20783
8	unidentified	*	0	null	1246.1	544.544	1.69614
(E)-2-hexenal-D	6,728-26-3	C_6_H_10_O	98.1	green, banana, fat	1229.7	518.426	1.51669
(E)-2-hexenal-M	6,728-26-3	C_6_H_10_O	98.1	green, banana, fat	1230.3	519.393	1.18612
1-Penten-3-ol	616–25-1	C_5_H_10_O	86.1	ethereal, green, tropical fruity	1162.3	420.759	0.94126
delta 3-carene-M	13,466–78-9	C_10_H_16_	136.2	citrus, lemon, woody	1163.1	421.859	1.21477
delta 3-carene-D	13,466–78-9	C_10_H_16_	136.2	citrus, lemon, woody	1169.3	430.710	1.67375
Butanol	71–36-3	C_4_H_10_O	74.1	wine	1150.8	404.724	1.18339
beta-Thujene-M	28,634–89-1	C_10_H_16_	136.2	null	1123.8	369.676	1.21936
beta-Thujene-D	28,634–89-1	C_10_H_16_	136.2	null	1126.0	372.383	1.6369
beta-Thujene-T	28,634–89-1	C_10_H_16_	136.2	null	1127.4	374.187	2.1779
1-hexanal	66–25-1	C_6_H_12_O	100.2	fresh, green, fat, fruity	1091.7	331.782	1.27099
7	unidentified	*	0	null	1045.6	290.069	0.94508
Ethanol	64–17-5	C_2_H_6_O	46.1	aromaticity	950.6	222.962	1.13791
Dimethyl sulfide	75–18-3	C_2_H_6_S	62.1	cabbage, sulfur, gasoline	788.7	158.914	0.95985
Ethylene glycol	107–21-1	C_2_H_6_O_2_	62.1	odorless	1665.1	1342.486	1.09549
2-Undecanone	112–12-9	C_11_H_22_O	170.3	wax, fruity, cream, fat, iris	1609.6	1198.476	1.54807
d-camphor	464–49-3	C_10_H_16_O	152.2	cool, pungent, strong medicinal taste	1514.7	987.039	1.35124
Decanal	112–31-2	C_10_H_20_O	156.3	sweet, waxy, floral, citrus, aldehyde, fatty	1500.6	958.904	1.53837
1	unidentified	*	0	null	1428.3	827.084	1.23340
(E)-Ethyl-2-hexenoate-D	27,829–72-7	C_8_H_14_O_2_	142.2	fruity, vegetable	1336.7	685.730	1.80761
(E)-Ethyl-2-hexenoate-M	27,829–72-7	C_8_H_14_O_2_	142.2	fruity, vegetable	1337.2	686.408	1.31821
1-hexanol	111–27-3	C_6_H_14_O	102.2	fresh, fruity, wine, sweet, green	1373.5	739.309	1.32574
2-Octanone	111–13-7	C_8_H_16_O	128.2	mouldy, ketone, milk, cheese, mushroom	1299.3	635.203	1.33327
1-Pentanol	71–41-0	C_5_H_12_O	88.1	balsamic	1263.1	572.92	1.24949
2	unidentified	*	0	null	1243.4	540.204	1.21422
(+)-limonene-M	138–86-3	C_10_H_16_	136.2	lemon, sweet, orange, pine oil	1201.3	476.179	1.21422
(+)-limonene-D	138–86-3	C_10_H_16_	136.2	lemon, sweet, orange, pine oil	1201.5	476.531	1.65832
(Z)-2-Hexenal	16,635–54-4	C_6_H_10_O	98.1	null	1212.9	493.065	1.18418
delta 3-carene-T	13,466–78-9	C_10_H_16_	136.2	citrus, lemon, woody	1167.0	427.434	2.15747
3	unidentified	*	0	null	1153.5	408.418	1.49871
Allyl sulfide	592–88-1	C_6_H_10_S	114.2	garlic	1153.4	408.278	1.12582
beta-Pinene-M	127–91-3	C_10_H_16_	136.2	resin, green	1104.5	346.416	1.21353
beta-Pinene-D	127–91-3	C_10_H_16_	136.2	resin, green	1108.0	350.527	1.63851
beta-Pinene-T	127–91-3	C_10_H_16_	136.2	resin, green	1111.8	355.039	2.11966
alpha-Fenchene	471–84-1	C_10_H_16_	136.2	null	1065.4	307.052	1.21113
alpha-Pinene-D	80–56-8	C_10_H_16_	136.2	fresh, camphor, sweet, pine wood	1026.6	274.590	1.66574
alpha-Pinene-M	80–56-8	C_10_H_16_	136.2	fresh, camphor, sweet, pine wood	1021.4	270.513	1.21279
4	unidentified	*	0	null	1021.4	270.548	1.11463
3-Pentanone-D	96–22-0	C_5_H_10_O	86.1	ethereal	994.2	250.204	1.35448
3-Pentanone-M	96–22-0	C_5_H_10_O	86.1	ethereal	992.9	249.251	1.11164
2-Butanone	78–9-33	C_4_H_8_O	72.1	fruity, camphor	917.9	204.783	1.24502
2-Propanol	67–63-0	C_3_H_8_O	60.1	alcohol, spicy	933.0	212.958	1.22595
Methyl acetate	79–20-9	C_3_H_6_O_2_	74.1	ethereal	850.4	179.294	1.03116
2-propanone	67–64-1	C_3_H_6_O	58.1	fresh, apple, pear	829.9	172.240	1.1182
5	unidentified	*	0	null	945.8	220.172	1.17827
Methyl propanoate	554–12-1	C_4_H_8_O_2_	88.1	Fruit, Rum	932.3	212.600	1.32243
1-Methyl-1H-pyrrole-2-carboxaldehyde	1,192-58-1	C_6_H_7_NO	109.1	nut	1624.6	1235.900	1.12403
6	unidentified	*	0	null	1565.9	1096.044	1.42923
Cyclohexanone	108–94-1	C_6_H_10_O	98.1	strong pungent, earthy	1300.4	636.578	1.15688
Dimethyl trisulfide	3,658-80-8	C_2_H_6_S_3_	126.3	fresh onion, mint, spicy	1392.4	768.498	1.30104
2-Heptanone	110–43-0	C_7_H_14_O	114.2	pear, banana, fruity, slight medicinal fragrance	1189.1	459.163	1.26452
Camphene	79–92-5	C_10_H_16_	136.2	woody, camphor	1081.1	321.135	1.21355

The substance suffixes M, D, or T represent the monomer, dimer, and trimer of the same substance, respectively, and numbers indicate unidentified peaks; The odor descriptions of each compound mainly referenced https://www.flavornet.org, https://www.femaflavor.org, and https://www.chemicalbook.com.

**Figure 5:**
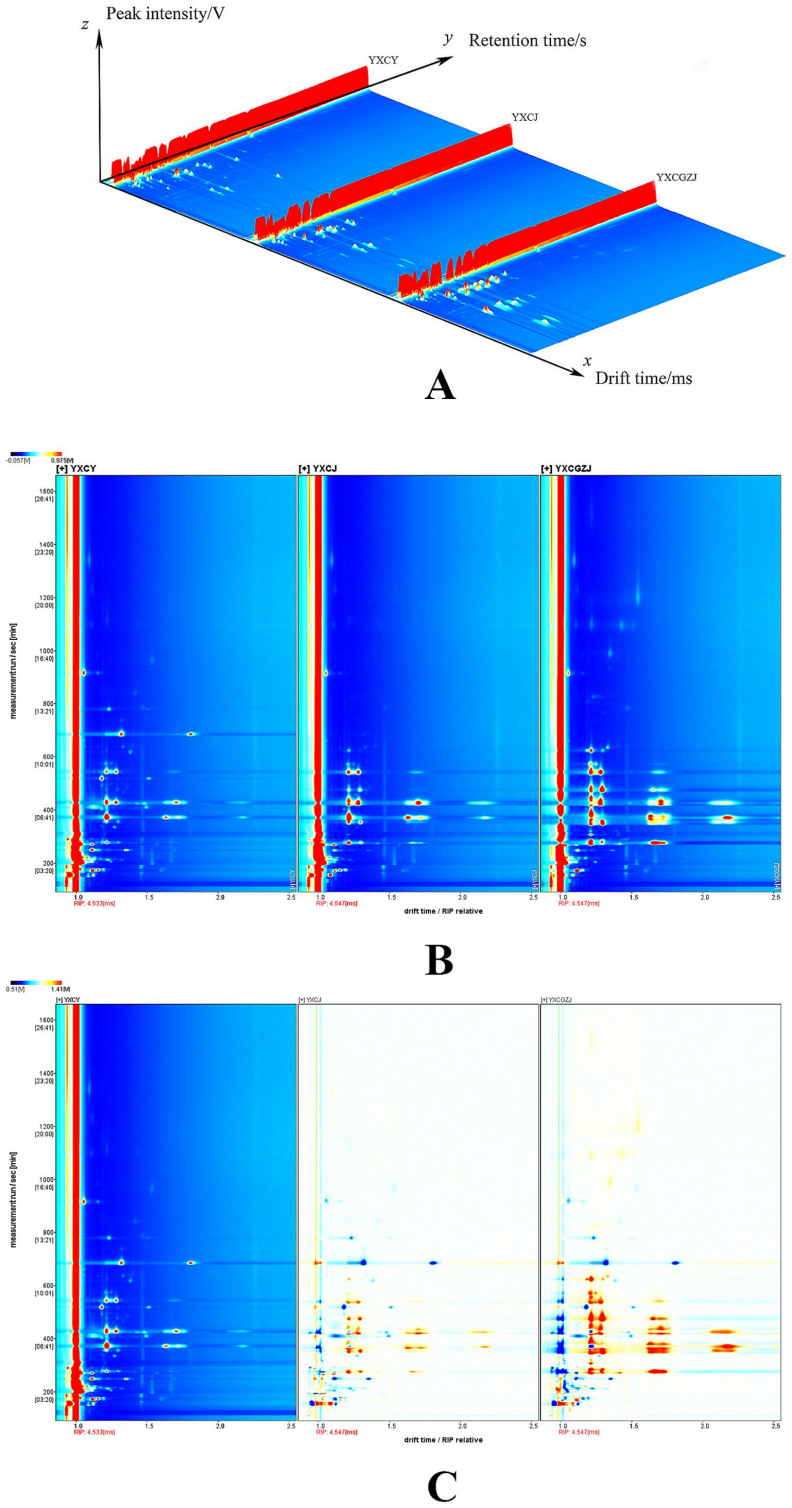
3D spectrum **(A)**, 2D spectrum **(B)** and comparative spectrum **(C)** of VOCs from different parts of *H. cordata*.

The VOCs from the samples generated a 3D spectrogram, where the three axes (X, Y, and Z axes) stand for ion drift time, retention time, and signal peak intensity, as shown in Figure 5A. The corresponding top view is shown in Figure 5B, featured a blue background with axes indicating retention time and relative migration time. A red vertical line at position 1.0 represented a normalized Reaction Ion Peak (RIP). Each point on either side of RIP represents a VOC, and the color intensity indicated their peak intensity. By using the YXCY spectrogram as the reference, a differential comparison map was generated by subtracting the other spectrograms, as depicted in Figure 5C. White indicated identical peak intensity, while red and blue signify levels above and below the reference, respectively. These results demonstrate significant variations in VOCs peak intensity among the rhizomes, stems, and leaves of *H. cordata*.

#### 3.2.2 Analysis of fingerprint

**Figure 6:**
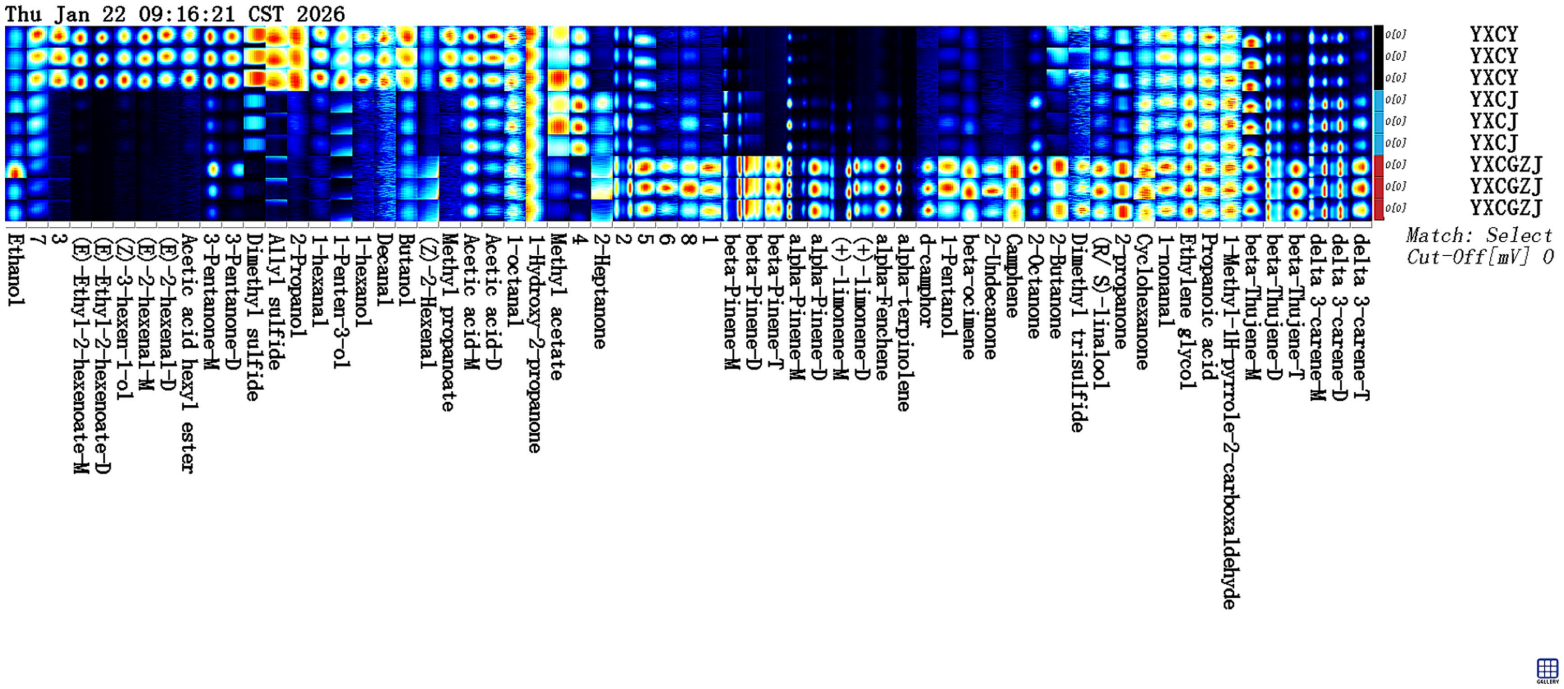
Fingerprint spectrum of VOCs from different parts of *H. cordata*.

To visually compare VOCs differences in different parts of *H. cordata*, we used the Gallery Plot Plugin to extract peaks from GC-IMS spectra and created a fingerprint spectrum (Figure 6). Each row represents all signal peaks for a sample, while each column represents the same VOCs in different samples. Color intensity indicates peak intensity, with red for high and blue for low. The results revealed that (E)-Ethyl-2-hexenoate, (Z)-3-hexen-1-ol, (E)-2-hexenal, Acetic acid hexyl ester, 1-Penten-3-ol, 1-hexanal, Decanal, (Z)-2-Hexenal, Methyl propanoate, 3-Pentanone, Dimethyl sulfide, Allyl sulfide, 2-Propanol, 1-hexanol, Butanol, Acetic acid, 1-octanal, 1-Hydroxy-2-propanone are highly relative abundance in the leaves of *H. cordata* (YXCY), and the first nine VOCs are unique to this part. Beta-Pinene, (+)-limonene, d-camphor, 2-Undecanone, Camphene, Dimethyl trisulfide, alpha-Pinene, alpha-Fenchene, alpha-terpinolene, 1-Pentanol, beta-ocimene, 2-Octanone, 2-Butanone, (R/S)-linalool, 2-propanone, Cyclohexanone, 1-nonanal, Ethylene glycol, Propanoic acid, beta-Thujene, 1-Methyl-1H-pyrrole-2-carboxaldehyde, delta 3-carene are highly relative abundance in the rhizomes of *H. cordata* (YXCGZJ), and the first six VOCs are unique to this part. 2-Heptanone is high relative abundance in the stems of *H. cordata* (YXCJ), and it is a component exclusive to this part.

#### 3.2.3 PCA and ED

**Figure 7:**
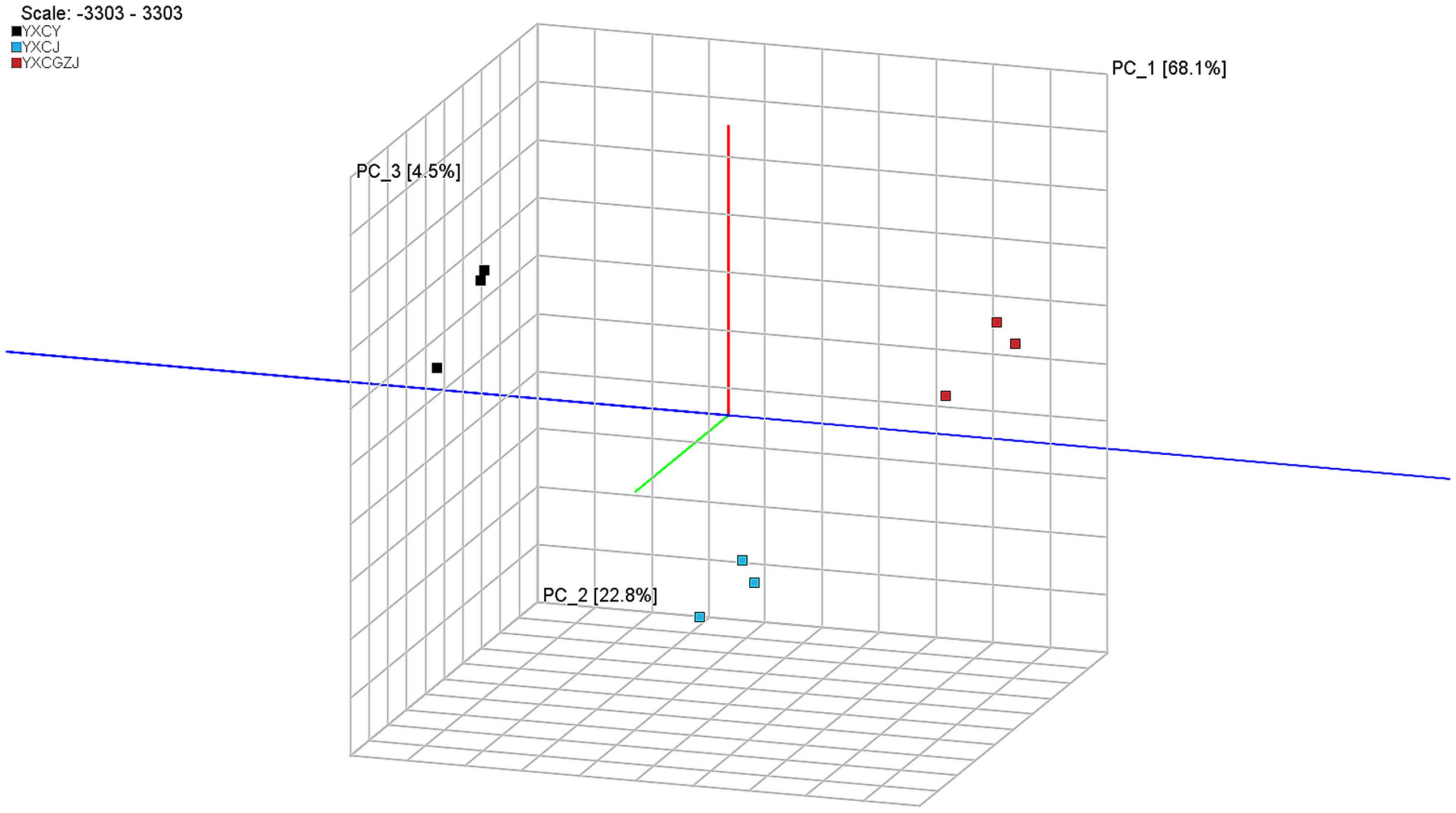
PCA 3D scatter plot of VOCs from different parts of *H. cordata*.

**Figure 8:**
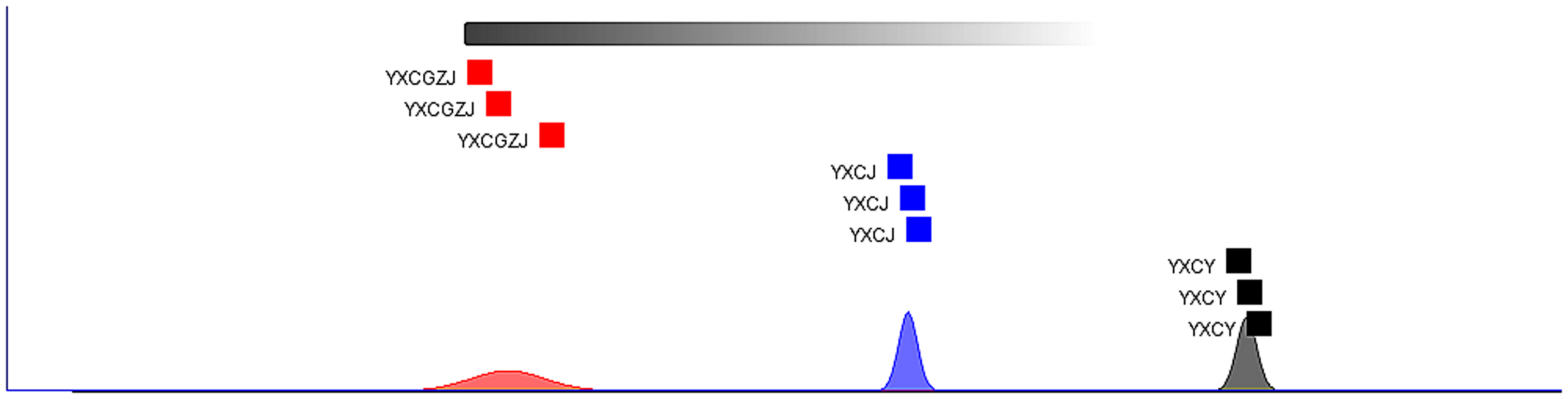
Fingerprint similarity based on ED from different parts of *H. cordata*.

To clearly differentiate between the leaves, stems, and rhizomes of *H. cordata*, PCA was performed on the peak intensity of VOCs from the samples, as shown in Figure 7. PC1 contributed 68.1%, while PC2 contributed 22.8%, and the cumulative contribution rate reached 90.9%. The PCA score plot revealed that replicate samples clustered within their respective groups, while distinct groups were completely separable, indicating significant differences in VOCs across different parts, particularly between the leaves and rhizomes. Furthermore, ED analysis showed the distances between different samples could be clearly distinguished, and the results are shown in Figure 8. These results indicated significant differences in VOCs profiles among the YXCY, YXCJ, and YXCGZJ samples, which could be clearly distinguished from one another. Among them, leaves and rhizomes showed the greatest separation, demonstrating the most substantial difference between these two parts. This pattern aligned with the observations from the PCA analysis.

#### 3.2.4 PLS-DA and CA

**Figure 9:**
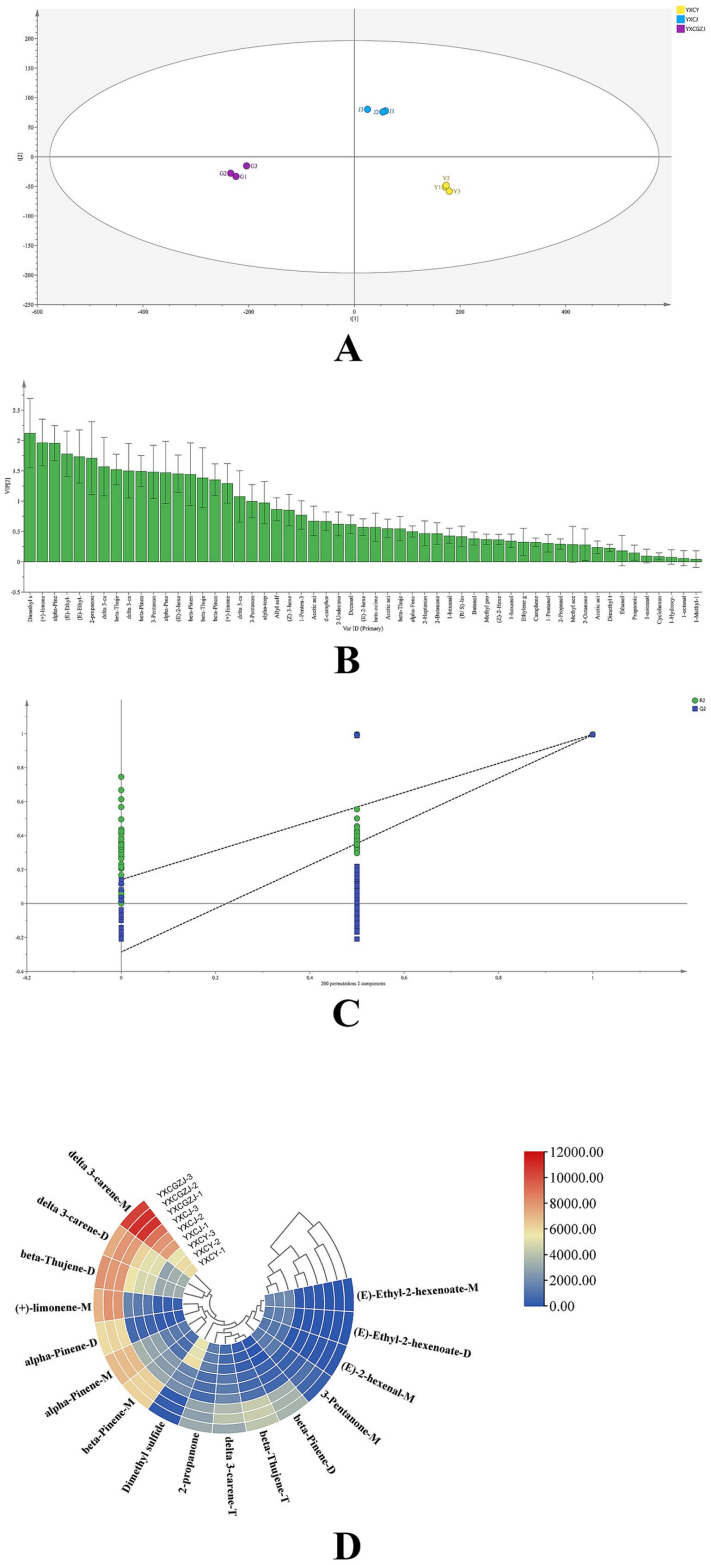
PLS-DA **(A)**, VIP values **(B)**, permutation test **(C)** and cluster heat map **(D)** of VOCs from different parts of *H. cordata*.

To distinguish the three parts of *H. cordata*, the peak intensity results of 55 VOCs from different samples were normalized, and PLS-DA was performed using SIMCA 14.1. The results are shown in Figure 9A, where Q^2^ = 0.991 > 0.5, R^2^X = 0.984 and R^2^Y = 0.994. The three sample groups are well separated and have good differentiation, indicating significant differences in VOCs peak intensity, which is consistent with the results of PCA and ED.

Variable importance in projection (VIP) was used evaluate the correlation between metabolites and sample classification. When VIP score exceeding 1 can be considered potential characteristic VOCs. As shown in Figure 9B, there are 16 VOCs with VIP > 1 and *p* < 0.05, ranked by their VIP values are Dimethyl sulfide, (+)-limonene-M, alpha-Pinene-D, (E)-Ethyl-2-hexenoate-M, (E)-Ethyl-2-hexenoate-D, 2-propanone, delta 3-carene-M, beta-Thujene-T, delta 3-carene-D, 3-Pentanone-M, alpha-Pinene-M, (E)-2-hexenal-M, beta-Pinene-M, beta-Thujene-D, beta-Pinene-D and delta 3-carene-T. To avoid overfitting, we conducted 200 random permutations of group labels using SIMCA to examine R^2^ and Q^2^ values. The steep regression line in Figure 9C indicates that the PLS-DA model was not overfitting (R^2^ = 0.14; Q^2^ = −0.286), The findings indicated that the model exhibited satisfactory predictive capability.

To evaluate whether 16 VOCs could serve as potential characteristic VOCs for the three parts of *H. cordata*, a clustering heatmap was generated from their peak intensity. Colors ranged from blue (low abundance) to red (high abundance), as shown in Figure 9D. The results aligned with those from PCA and PLS-DA analyses. In the rhizomes of *H. cordata*, delta 3-carene-M, beta-Thujene-D, delta 3-carene-D, beta-Pinene-M, alpha-Pinene-M, alpha-Pinene-D, (+)-limonene-M, delta 3-carene-T, beta-Pinene-D and beta-Thujene-T, were more relative abundant than in stems and leaves. Dimethyl sulfide was significantly higher in leaves compared to rhizomes and stems. Notably, delta-3-carene-M, beta-Thujene-D and delta-3-carene-D differed markedly between the leaves and stems, enabling their discrimination. The heatmap further revealed that the three parts formed two distinct clusters: rhizomes alone in one cluster, and leaves and stems together in the other. This grouping indicates that leaves and stems share similar VOCs profiles. These findings were consistent with the results from GC-IMS fingerprinting, PCA, and PLS-DA. Therefore, the 16 VOCs can effectively distinguish the components of different parts of *H. cordata* and serve as potential characteristic VOCs for their identification.

## 4 Discussion

Volatile odor serves as crucial sensory indicator intimately linked to the quality and therapeutic effects of traditional Chinese medicine. A direct and highly significant correlation exists between odor and VOCs. From a chemical perspective, the aromas of traditional Chinese medicines are composed of various VOCs, and variations in the types and quantities of these compounds can result in different odor profiles (22). Traditional sensory relies heavily on the expertise of experienced pharmacists, which renders the process inherently subjective. Moreover, inter-individual differences in olfactory sensitivity and susceptibility to olfactory fatigue pose significant challenges to the objective quantification of odor characteristics (23). The conventional “nose-sniffing” method for identifying the essence of traditional Chinese medicines is based on subjective perception of the VOCs spectrum. However, the integration of modern analytical techniques has facilitated the objective evaluation of traditional Chinese medicines aroma, transforming traditional sensory identification into a scientifically rigorous analysis grounded in VOCs profiles. This objectification of the traditional practice of “assessing by smell and taste” offers significant technical support for traditional Chinese medicines and enhances research into its therapeutic efficacy.

Currently, relying on a single analytical method may lead to the omission of key compounds, whereas the combination of multiple techniques provides a more comprehensive and reliable dataset (24). This study innovatively combines the rapid screening advantages of E-nose with the high-resolution qualitative and quantitative capabilities of GC-IMS. It systematically examined the variations in VOCs present in the rhizomes, stems, and leaves of *H. cordata*, thereby providing a chemical basis for part-specific identification and resource utilization. Notably, the distinct detection principles and incompatible data formats of these technologies preclude direct numerical comparison. GC-IMS, equipped with the polar MXT-WAX column, precisely quantifies trace polar flavor compounds. This analytical specificity enables mechanistic interpretation of flavor chemistry. However, this method does not account for multi-component olfactory synergy and fails to capture sensory-level odor profiles. In contrast, the E-nose, employing the weakly polar MXT-5 and moderately polar MXT-1701 columns, which can rapidly acquire holistic odor fingerprints that align with human olfactory perception. But it is unable to distinguish individual differential marker compounds. Collectively, GC-IMS targets the analysis of microscopic volatile compounds, whereas the E-nose is designed to capture macroscopic odor profiles. The two technological instruments are complementary; when used in conjunction, they provide a comprehensive evaluation of odor and VOCs.

The Heracles Neo Ultra-Fast Gas Phase Electronic Nose, using dual capillary columns (MXT-5/MXT-1701) with FID, analyzed the overall odor profiles of VOCs from different parts of *H. cordata* in 5 min. The leaves of *H. cordata* are rich in alcohols and aldehydes, such as 1-Octanethiol and Decanal. In contrast, its rhizomes have higher levels of alkanes and terpenoids, such as 3-Ethyloctane, Tetraethoxysilane, 1R- (+)-alpha-pinene, and gamma-Terpinene. The types and relative abundances of VOCs in the stems are intermediate. The PCA, DFA, and radar chart results of the E-nose indicated that the aroma of different parts of *H. cordata* existed obvious differences. These findings are consistent with the PCA and ED results of GC-IMS, confirming that variations in VOC composition are fundamental drivers of the distinct odors associated with different parts of *H. cordata*.

Although the E-nose can effectively differentiate the overall odor characteristics of different parts of *H. cordata* due to its sensor array, it is challenging to apply it for qualitative and quantitative analysis of VOCs. To overcome this limitation, this study systematically analyzed the VOCs composition in the rhizomes, stems, and leaves of *H. cordata* by combining GC-IMS technology. A total of 63 signal peaks were detected, and 55 VOCs were identified, including 16 terpenes (29.091%), 9 ketones (16.364%), 9 alcohols (16.364%), 8 aldehydes (14.545%), and 5 esters (9.091%). Terpenes, the most abundant VOCs in *H. cordata*, are not only the key source of its refreshing woody aroma but also possess antibacterial, anti-inflammatory, and antioxidant activities (25). Ketones, alcohols, and aldehydes are slightly less abundant and mainly responsible for its pungent odor. For instance, 1-hydroxy-2-propanone and cyclohexanone produce irritating odors, while 2-propanol contributes a spicy note. Esters, present at lower relative abundances, primarily function as flavor modifiers with relatively minor contributions to the overall bioactivity.

In order to compare the peak intensity of VOCs from different parts of *H. cordata,* we conducted the analysis using PCA and ED. The results show that there are significant differences among them, which may be related to the distinct growth locations, developmental stages, and physiological functions inherent in *H. cordata*. Using PLS-DA and CA, 16 VOCs with VIP values greater than 1 and *p*-values below 0.05 were identified and designated as potential characteristic volatile components for distinguishing the different parts of *H. cordata*. These compounds can provide important guidance for the identification and utilization of the various parts of *H. cordata*. Notably, the rhizomes of *H. cordata* contain high relative abundances of four monoterpene compounds: 3-carene (26–28), beta-thujene (29), pinene (30, 31), and limonene (32, 33). These ingredients not only impart a distinctive fragrance, for instance 3-carene is known for its volatility and characteristic aroma, making it a common ingredient in food flavoring, but also via synergistic effects, including anti-inflammatory, antibacterial and antioxidant properties, which collectively confer a “herbal antibiotic” function within the rhizome. The volatile composition of the stems closely resembles that of the rhizomes, with differences primarily in peak intensity, leading to a milder aroma. This characteristic renders the stems suitable not only as a fresh sprout vegetable or functional food ingredient, but also for consumers who prefer a subtler flavor profile while still seeking antibacterial and antiviral benefits. In contrast to the rhizomes and stems, the leaves are distinguished by a high signal response of Decanal. Decanal functions as a “natural weapon” for plants, conferring defense against fungal infections and demonstrating exceptional efficacy in disrupting the cellular structure of pathogenic bacteria (34, 35). Consequently, we speculate that the rhizomes of *H. cordata,* which accumulate abundant antibacterial and anti-inflammatory volatiles, have potential as candidate materials for future development of related pharmaceutical products. While its stems possess f distinct volatile flavor characteristics, suggesting potential for health-promoting functional foods. The leaves, enriched in antimicrobial and pest-resistant volatiles, are promising candidates for potential eco-friendly pesticides and natural antimicrobials. Notably, these application prospects are speculative and derived only from their differential enrichment of bioactive VOCs and reported compound bioactivities in different parts of *H. cordata*. Consequently, further validation of bioactivity, comprehensive safety evaluations, and feasibility studies are crucial to confirm their practical applicability.

## 5 Conclusion

This study utilized GC-IMS and the Heracles Neo electronic nose to analyze and identify VOCs in various parts of *H. cordata*. The findings revealed distinct odors and VOCs profiles across different parts, with leaves and stems exhibiting the greatest similarity. A practical and scientifically rigorous rapid identification system for differentiating the parts of *H. cordata* was established. This integrated approach demonstrates high accuracy and efficiency, enabling convenient and rapid identification of different parts of *H. cordata*. However, this study has limitations: the existing standard database of GC-IMS is not perfect, limiting the accurate quantitative analysis and comprehensive characterization of samples; E-nose lack specificity and are easily disrupted by substrates, affecting odor recognition and component discrimination accuracy. Therefore, our next step is to utilize other advanced analytical techniques, such as GC–MS and GC-O, to further verify and deeply analyze the VOCs of *H. cordata.* Future research will focus on the differences in VOCs and odor characteristics of different parts of *H. cordata* from different origins and at different growth stages. This will provide a more comprehensive theoretical basis for enhancing the resource utilization of *H. cordata*.

## Data Availability

The original contributions presented in the study are included in the article/supplementary material, further inquiries can be directed to the corresponding author.
